# Non-pharmacological treatment of MASLD

**DOI:** 10.1007/s00125-026-06811-5

**Published:** 2026-07-25

**Authors:** Jean-François Dufour, Annalisa Berzigotti

**Affiliations:** 1 Centre for Digestive Diseases, Lausanne, Switzerland; 2 Department of Visceral Surgery and Medicine, Bern University Hospital, University of Bern, Bern, Switzerland

**Keywords:** Cardiometabolic health, Diet, Exercise, Lifestyle intervention, Metabolic dysfunction-associated steatotic liver disease (MASLD), Non-pharmacological treatment, Physical activity, Review, Sleep optimisation, Weight loss

## Abstract

Non-pharmacological treatment of metabolic dysfunction-associated steatotic liver disease (MASLD) aims at modifying lifestyle patterns that promote metabolic dysfunction and hepatic fat accumulation. These include unhealthy dietary habits, which are characterised by excess energy intake and a qualitatively poor diet that is high in ultra-processed food, saturated fat, free sugar, red and processed meat and/or sweetened beverages, and low in fibre. In addition, sedentary habits, and sleep and circadian rhythm disruption have been identified as detrimental lifestyle factors. Lifestyle interventions act upstream of the main pathophysiological mechanisms driving MASLD and, therefore, provide benefits that extend beyond the liver, also improving cardiometabolic health. Weight loss remains the cornerstone of non-pharmacological strategies, and even modest weight loss has been proven beneficial in individuals with MASLD with or without obesity. To achieve weight loss, energy restriction should be combined with physical activity/exercise, since the effects of the latter are partly independent of weight reduction. Importantly, exercise is associated with a dose-dependent reduction in the risk of hepatic steatosis, fibrosis and hepatocellular carcinoma. Despite lifestyle changes proving highly effective for improving MASLD, their implementation is challenging and requires multidisciplinary efforts. Adherence is suboptimal, particularly in the long term, and the variability in individual response calls for research on biomarkers to achieve a more personalised approach. This review provides an overview of evidence of the impact of lifestyle changes/non-pharmacological interventions on MASLD.

**Graphical Abstract:**

**Figure:**
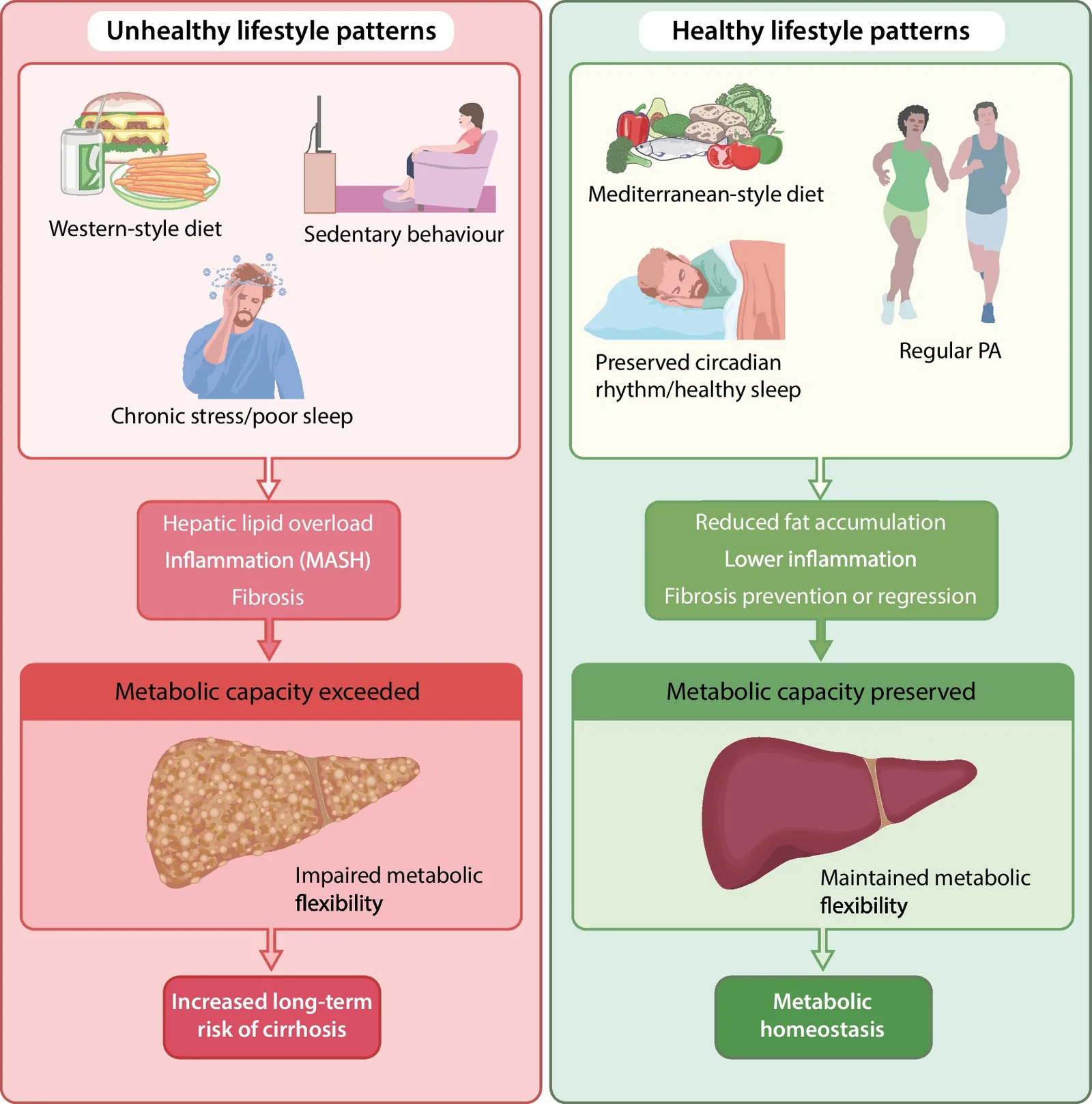

**Supplementary Information:**

The online version contains a slideset of the figures for download available at https://doi.org/10.1007/s00125-026-06811-5.

## Introduction

Metabolic dysfunction-associated steatotic liver disease (MASLD), which was previously termed ‘non-alcoholic fatty liver disease’ (NAFLD) or ‘metabolic dysfunction-associated fatty liver disease’ (MAFLD), has steadily increased in the general population in recent decades. As a result, it is currently very common, with an estimated global prevalence of 38% in the adult population [1] and of 7–14% in children and adolescents [2]. While non-modifiable predisposing factors to MASLD have been described, which include genetic variants (e.g. *PNPLA3*), in utero and early-life exposure to under- or overnutrition, and sex- and ageing-related factors, physicians should recognise that the cardiometabolic risk factors driving disease onset are largely modifiable through lifestyle intervention.

These factors include the components of the metabolic syndrome, namely visceral (central) obesity, insulin resistance, dyslipidaemia and arterial hypertension. Among them, obesity-driven adipose tissue dysfunction and chronic low-grade inflammation play a central role in the development of insulin resistance and MASLD. Therefore, while assessment and treatment of MASLD should be seen as a holistic effort to identify and address all factors driving the disease, a large body of evidence supports the prioritisation of reduction (in terms of mass) and remodelling of adipose tissue as central therapeutic targets in MASLD [3]. To address this, it is important to understand the factors leading to and sustaining obesity.

Causes of the sharp increase in obesity worldwide [4] have been detailed elsewhere [5]. A sustained positive energy balance (energy intake exceeding expenditure) is the central cause of weight gain [5]. This may result from the convergence of biological factors (primarily the dysregulation of appetite and energy homeostasis [leptin resistance, impaired satiety signalling]), environmental and behavioural drivers (food environment, eating patterns, physical activity [PA] opportunities), and socioeconomic and societal structures (food access, work demands, health literacy). These factors interact dynamically across the lifespan, with early-life exposures (maternal nutrition, childhood feeding patterns, socioeconomic stress) shaping lifelong vulnerability to energy imbalance and metabolic dysfunction [5]. Disrupted appetite/satiety can be targeted by incretins [6], which have consistently been proven to induce weight loss and improve MASLD. However, non-pharmacological interventions provide benefits that go beyond weight loss and remain the first line treatment option [3].

This review provides an integrated overview of non-pharmacological MASLD management, analysing the effects achieved through weight loss and those that are likely to be dependent on metabolic- or microbiota-mediated mechanisms. In brief, factors implicated in MASLD onset and progression include sustained positive energy balance, excessive intake of energy-dense, ultra-processed foods with high sugar content and saturated fat, free fructose-loaded drinks, and insufficient energy expenditure owing to a sedentary lifestyle (these factors are addressed in more detail below). We also provide an overview of emerging evidence that supports a role for sleep disruption, chronic psychological stress [7] and environmental exposures (including air pollution) [8] as contributing factors leading to MASLD.

## Pathophysiological rationale for lifestyle interventions

MASLD is defined by the presence of excess storage of triacylglycerols in the liver (in ≥5% of hepatocytes) alongside at least one cardiometabolic risk factor. Its severity varies from simple steatosis, which can conceptually be considered as an asymptomatic stage linked to increased cardiometabolic- and liver-related risks [9], to overt liver disease (steatohepatitis, MASH), characterised by hepatocellular injury, inflammation and fibrosis. Inflammation and fibrosis are associated with an aggressive disease course, with progression to advanced chronic liver disease with portal hypertension and the development of hepatocellular carcinoma in a subset of individuals [3].

### Pathophysiology of MASLD

Hepatic triacylglycerol accumulation (steatosis) in MASLD results from an imbalance in lipid influx, synthesis and disposal [10, 11]. Sustained excess energy, leading to ectopic fat accumulation, is the central driver. In individuals with obesity, excess energy intake and adipose tissue dysfunction lead to an excessive influx of non-esterified fatty acids in the liver. These are then esterified into triacylglycerols in the liver or transformed into lipid intermediates (e.g. diacylglycerols, ceramides) that promote lipotoxicity. In addition, insulin resistance in skeletal muscle (primarily caused by residual glucose that is unable to be stored post-prandially) [12], adipose tissue and the liver leads to increased de novo lipogenesis and impaired hepatic fatty acid oxidation (owing to mitochondrial dysfunction). This is partly mediated by a high-fat diet and hyperglycaemia-associated induction of transcription factors, such as sterol regulatory element‐binding protein 1 (SREBP‐1) and carbohydrate response element‐binding protein (ChREBP) [13, 14]. Moreover, reduced export of triacylglycerols via very-low-density lipoproteins is a hallmark of MASH.

Lipotoxicity triggers endoplasmic reticulum stress, mitochondrial dysfunction and oxidative stress. Kupffer cells and monocyte-derived macrophages are successively activated and promote hepatic stellate cell activation and extracellular matrix deposition, leading to fibrosis. In addition to the central role of excessive energy intake, it is proposed that low-grade inflammation, originating from the adipose tissue, further amplifies this cascade [15–17]. Furthermore, saturated fat, refined carbohydrates and ultra-processed industrial food promote gut dysbiosis [18] and intestinal barrier dysfunction [19, 20]. This is followed by increased translocation of microbial proinflammatory products, such as lipopolysaccharide, further increasing systemic inflammation [21]. Western diet-induced gut dysbiosis modifies the composition of the bile acid pool by modifying microbial bile salt hydrolase activity and secondary bile acid production [22]. These changes lead to aberrant intestinal and hepatic signalling through bile acid-sensing receptors, particularly Farnesoid X receptor (FXR) and Takeda G-protein-coupled receptor 5 (TGR5) [23, 24]. Dysregulated FXR signalling disrupts feedback control of bile acid synthesis and impairs hepatic lipid and glucose metabolism by promoting de novo lipogenesis and hepatic insulin resistance, while altered TGR5 activation reduces energy expenditure and increases inflammatory response [24].

### Lifestyle interventions target MASLD drivers

**Fig. 1:**
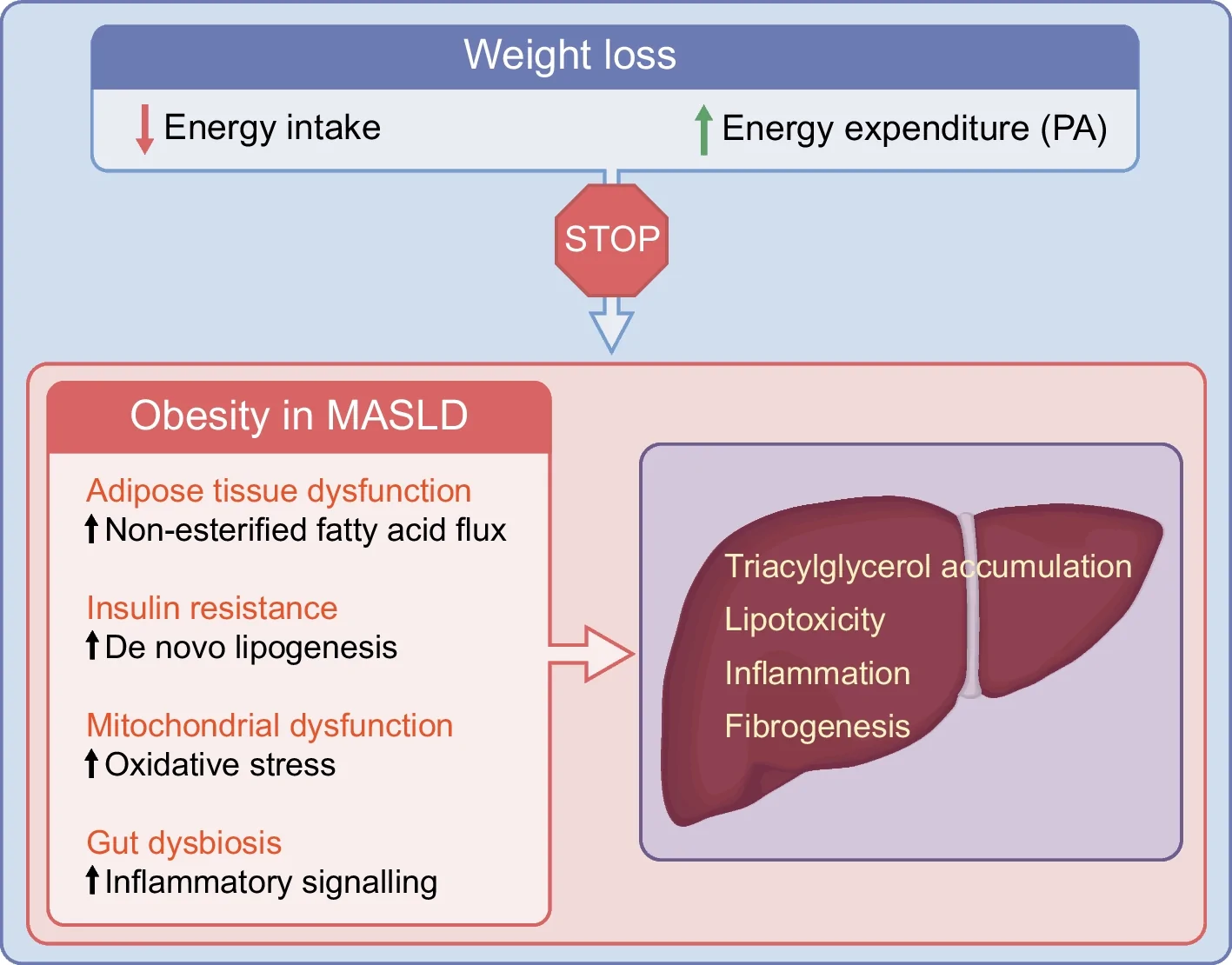
Impact of weight loss on the main pathophysiological mechanisms driving MASLD. Adipose tissue dysfunction, insulin resistance, mitochondrial dysfunction and gut dysbiosis converge to induce hepatic triacylglycerol accumulation, lipotoxicity, inflammation and fibrogenesis. Weight loss achieved through energy restriction and PA, act upstream of these mechanisms to favourably modify the course of MASLD. This figure is available as part of a downloadable slideset

Lifestyle interventions have the potential to prevent and favourably modify the course of MASLD by targeting several main drivers, namely insulin resistance, adipose tissue dysfunction, mitochondrial impairment and gut dysbiosis. Since obesity is an upstream determinant of the aforementioned pathophysiological changes in MASLD, weight loss (achieved through diet and PA/exercise) remains the cornerstone of MASLD management (Fig. 1).

In general, weight reduction improves insulin sensitivity and reduces chronic low-grade inflammation. Beyond its liver-specific effects, weight loss favourably influences cardiometabolic risk factors, including glycaemic management, dyslipidaemia and blood pressure, so improving overall cardiometabolic health.

## Clinical aspects of MASLD: weight loss as the central therapeutic target

### Weight loss

Weight loss is the foundation of MASLD management. It has been proven that even modest weight loss reduces intrahepatic fat content, and a dose–response relationship has been observed between weight loss and liver inflammation, hepatocyte ballooning and resolution of MASLD/MASH [25, 26]. The joint recommendations of the European Association for the Study of the Liver (EASL), the European Association for the Study of Diabetes (EASD) and the European Association for the Study of Obesity (EASO) [3], published in 2024, state that a weight reduction of ≥5% is sufficient to decrease hepatic fat content, while losses of 7–10% are associated with improvements in hepatic inflammation and of ≥10% may lead to fibrosis regression. Importantly, even in adults with MASLD who are not overweight or obese but have (as per definition) at least one cardiometabolic risk factor, a modest weight loss of 3–5% improves liver-related outcomes.

Furthermore, MASLD can be abolished by weight loss of more than 5% with concomitant return to normal blood glucose levels in type 2 diabetes [27–29].

### Energy restriction

In a recent meta-analysis that included 60 studies and over 100,000 individuals [30], total energy intake was clearly associated with the presence of MASLD. Hence, energy restriction should also be considered an important lifestyle change for the management of MASLD. Pathophysiologically, energy restriction improves mitochondrial function and metabolic flexibility in both the liver and skeletal muscle [31]. In addition, reduced substrate overload alleviates mitochondrial stress, enhances β-oxidation efficiency, and lowers production of reactive oxygen species [32]. Autophagy pathways, which clear excess lipid droplets and damaged cells, are also activated in conditions of energy restriction and contribute to the resolution of MASLD [33].

Clinical trials and meta-analyses consistently show that a daily energy deficit of approximately 2.1–4.2 MJ (500–1000 kcal), or a total energy intake within the range of 5.0–6.3 MJ per day (1200 kcal to 1500 kcal per day), are effective at promoting clinically significant weight loss and steatosis regression [34].

Standard approaches use hypoenergetic diets to induce continuous energy restriction (regular intake of meals during the day that are of better quality and smaller quantity than conventional diets). Intermittent energy restriction, either as intermittent fasting (>60% energy restriction on >2 days per week) or time-restricted feeding, has been proposed as an alternative to continuous energy restriction in MASLD. Across RCTs on MASLD/NAFLD, intermittent fasting strategies are, at least, as effective as continuous daily energy restriction for reducing liver fat, while often achieving a similar weight loss (Table 1). Some studies show slightly superior liver fat or fibrosis improvements with intermittent fasting regimens vs continuous/standard energy restriction, even when weight loss is comparable; however, these advantages are modest and short term. In our view, given the overall similarities of intermittent fasting, time-restricted feeding and continuous energy restriction, patient preference and adherence should guide the choice of energy restriction strategy adopted, with the important factor being achieving the necessary degree of weight loss.

**Table 1:** RCTs on intermittent energy restriction and time-restricted eating in MASLD/MAFLD

RCT author, year of publication	Population^a^	Design and groups	Duration	Primary outcome	Evidence quality^b^	Main findings vs continuous/standard energy restriction
Johari et al, 2019 [104]	43 adults with NAFLD	Modified alternate day energy restriction vs habitual diet	8 weeks	Steatosis grade, shear-wave elastography	Exploratory	Reduced BMI, ALT, steatosis and liver stiffness; good adherence to energy restriction diet
Wei et al, 2023 [105]	88 adults with obesity and NAFLD	TRE (08:00–16:00 hours) vs daily energy restriction	12 months	Intrahepatic fat content (assessed by MRI)	Robust	Similar reductions in liver fat, liver stiffness and weight
Sun et al, 2025 [106]	60 adults with MASLD and abnormal glucose metabolism	5:2 IER^c^ vs continuous energy restriction	12 weeks	Liver fat (assessed by ^1^H-MRS)	Robust	Similar reductions in liver fat and body weight
Wang et al, 2024 [107]	60 adults with MAFLD	5:2 IER^c^ vs daily energy restriction	12 weeks	Steatosis and fibrosis (assessed by CAP and LSM, respectively)	Exploratory	Similar weight loss; greater improvement in steatosis and fibrosis
Lee et al, 2025 [108]	72 participants without diabetes but with MASLD (MRI-PDFF ≥8%)	5:2 IER^c^ vs standard hypoenergetic diet	12 weeks	≥30% reduction in MRI-PDFF	Robust	Higher response rate with IER, especially in obesity
Oh et al, 2025 [109]	333 adults with MASLD, overweight/obesity	TRE vs energy restriction vs standard care^d^	16 weeks	Hepatic steatosis (assessed by MRI-PDFF)	Robust	TRE and energy restriction had similar ability to improve hepatic steatosis, but both superior to standard care
Nilghaz et al, 2025 [110]	53 adults with MAFLD	TRF 16:8^e^ + DASH diet vs low-energy control diet	12 weeks	Steatosis and fibrosis (assessed by CAP and LSM, respectively), liver enzymes	Exploratory	Greater improvements in steatosis, fibrosis and liver enzymes
Shafiee et al, 2025 [111]	42 adults with overweight/obesity and MAFLD	TRF 16:8^e^ + lacto-ovo-vegetarian diet vs control diet^f^	12 weeks	Weight and liver enzymes	Exploratory	Greater weight loss and larger improvements in liver enzymes
Ozlu Karahan et al, 2025 [112]	48 adults with overweight/obesity and MAFLD	TRF 16:8^e^ vs energy restriction	8 weeks	Steatosis and fibrosis (assessed by CAP and LSM, respectively), FGF-21, autophagy markers	Exploratory	Greater CAP reduction and autophagy markers

^a^The nomenclature used for NAFLD/MAFLD/MASLD is as reported in the articles

^b^Evidence quality was characterised as robust or exploratory

^c^5:2 intermittent energy restriction (also named 5:2 intermittent fasting) implies no specific energy restriction on 5 days of the week, followed by a substantial restriction of energy intake on 2 non-consecutive days; typically, energy is restricted to 2.09–2.51 MJ per day (500–600 kcal per day)

^d^Standard care included general advice about a healthy lifestyle but no specific structured dietary regimen

^e^TRF (or TRE) 16:8 implies that food intake is confined to an 8 h window each day, with the remaining 16 h spent fasting (consuming only water or energy-free beverages, such as black coffee or tea)

^f^Control diet: structured, mildly hypoenergetic standard weight-loss diet without any time restriction

CAP, controlled attenuation parameter; DASH, Dietary Approaches to Stop Hypertension; IER, intermittent energy restriction (also known as intermittent calorie restriction [ICR]); LSM, liver stiffness measurement; MRI, magnetic resonance imaging; PDFF, proton density fat fraction; TRE, time-restricted eating; TRF, time-restricted fasting

Studies conducted in individuals with type 2 diabetes have shown that liver fat content significantly decreases immediately after weight loss and that type 2 diabetes remission requires a decrease in liver fat content [28]. A very low-energy diet intervention of 8 weeks’ duration showed similar results to a standard diet (moderate energy restriction) in reducing hepatic fat content [35]. Interestingly, responders to the diet had sustained normalisation of fasting plasma glucose over the 6 months following the intervention [35].

### Qualitative dietary modifications

Quantitative changes in energy intake represent only one component of optimal nutritional management in MASLD. Beyond energy restriction, interventional studies have extensively examined qualitative dietary modifications as modulators of hepatic and systemic metabolic outcomes, particularly changes in food-intake habits (snacking) and in macronutrient composition of foods consumed. This is discussed in more detail below.

#### Dietary habits

Irregular/skipping breakfast and frequent snacking, particularly on energy-dense and high-glycaemic-load foods late at night, are associated with increased hepatic fat accumulation and insulin resistance, independent of total energy intake [36, 37]. While reducing snacking, especially late-day and nocturnal snacking, may suppress hepatic de novo lipogenesis and improve metabolic flexibility, direct evidence in MASLD remains limited.

#### Dietary patterns

Among the dietary patterns, the ‘Mediterranean’ diet is the most extensively studied and has proven to be associated with improvements in hepatic steatosis and liver enzymes and reduced risk of fibrosis progression [38]. In addition, it has proven beneficial for improving insulin resistance and reducing cardiovascular comorbidities. Qualitatively, the Mediterranean diet is rich in vegetables, legumes, fish and monosaturated fat (olive oil), and the food is minimally or unprocessed. This diet is rich in fibre and polyphenols, which favourably modulate the microbiome and bile acid profile. Current guidelines support this diet pattern in the management of MASLD [3].

#### Fat content

As for fat content, observational and interventional studies have shown that, independent of total energy intake, the quantity [39] and quality of dietary fat plays an important role in MASLD development. Higher intake of saturated fatty acids (SFAs) [40] and lower intake of polyunsaturated fatty acids (PUFAs) are associated with increased steatosis [40], while replacement of SFAs with monounsaturated fatty acids and PUFAs improves hepatic steatosis and insulin sensitivity, even in the absence of weight loss [41, 42]. It should be noted, however, that 100% of fat synthesised by de novo lipogenesis is saturated fat, and muscle insulin resistance determines fatty acid composition of the liver [43]. In experimental models, olive oil polyphenols exert antioxidant effects and modulate toll-like receptor signalling resulting in attenuation of hepatic inflammation and fibrogenesis [44]. In part, this likely explains the beneficial effects of Mediterranean diet. In addition, in several RCTs, dietary supplementation with *n*-3 fatty acids has been reported to decrease steatosis [45]; however, their effects on histological outcomes in MASLD are less clear.

#### Carbohydrate content

Low-carbohydrate diets (LCDs) have been evaluated as a strategy to reduce hepatic steatosis. In a meta-analysis, only a modest reduction in intrahepatic lipid content was found with LCD intake, with no consistent effects on liver enzymes and substantial heterogeneity across studies [46]. Some benefits of LCDs appear to be independent of weight loss, as isoenergetic, high-protein LCDs have been shown to reduce liver fat through increased hepatic fatty acid oxidation and suppression of de novo lipogenesis [47]. Ketogenic diets, a more restrictive form of LCD, can induce rapid short-term reductions in hepatic steatosis and improvements in insulin sensitivity, likely via enhanced lipid oxidation [48]. However, data are still limited, particularly in individuals with advanced liver disease, and long-term safety is unknown.

#### Sugar content

High fructose intake (including the consumption of free-fructose-loaded drinks [49]) is a well-identified factor that leads to the disruption of satiety and metabolic homeostasis and it induces hepatic steatosis per se [50]. Evidence suggests that fructose consumption contributes to MASLD [51] but whether it is sufficient to cause steatohepatitis in adults remains unclear [52]. Notably, the metabolic effects of fructose mimics those of alcohol [53]. In the paediatric population, consumption of fructose increases liver fat content [54], while the restriction of free sugars substantially reduces hepatic steatosis [55]. In a 4 week clinical trial, a fast food diet (which is strictly linked to high sugar consumption) was associated with significant weight gain, elevated alanine aminotransferase (ALT) and a doubling of liver fat content [56].

#### Protein content

High-protein diets, which are often embedded in hypoenergetic or low-carbohydrate regimens, have been associated with improvements in hepatic steatosis and liver enzymes in MASLD, although these effects are frequently confounded by concurrent weight loss [57]. In a small study, 37 individuals with concurrent diabetes and MASLD followed an isocaloric diet with high-protein intake for 6 weeks [58]. Dietary protein was either from animal- or plant-based sources. It was found that intrahepatic fat was reduced by 36–48%, with no negative metabolic effects of the diet containing animal-based proteins being detected [58]. On the other hand, red and processed meats have been linked to MASLD and insulin resistance [59]; hence, limited consumption of these types of protein is suggested.

#### Insoluble fibre intake

As a protective factor, in individuals with MASLD on a hypoenergetic diet, a higher insoluble fibre intake (≥7.5 g/day) was associated with improved non-invasive fat and fibrosis scores, as compared with individuals with an insoluble fibre intake of <7.5 g/day. These findings suggest that increased insoluble fibre intake might hold independent benefit to hypoenergetic diet alone [60].

#### Caffeine intake

In individuals with chronic liver disease within the general population, coffee consumption [61, 62] has been associated with a lower risk of MASLD (with an intake of more than 3 cups), and of fibrosis progression and hepatocellular carcinoma, [63]. Although, causal relationships remain uncertain in humans, in animal models of MASLD, caffeine has been shown to exert anti-inflammatory effects by reducing the expression of proinflammatory mediators in adipose tissue, and by attenuating hepatic inflammation and de novo lipogenesis [64]. These effects are accompanied by reductions in fat mass and improvements in systemic and hepatic insulin sensitivity.

To summarise, energy reduction through any of the diets discussed above (low-carbohydrate, low-fat, intermittent fasting and time-restricted eating patterns) can reduce liver fat. These diets are likely to be equivalent with regard to effectiveness if they produce an equivalent energy deficit. Therefore, patient preference to achieve optimal adherence should be carefully taken into consideration, keeping necessary weight loss to improve MASLD as the main target. For people with MASLD and type 2 diabetes, diets with a lower glycaemic load may also offer additional benefits for glycaemic management.

## PA and exercise with or without weight loss

PA is a key modulator of hepatic fat accumulation and plays a central role in the pathophysiology of liver steatosis. Lower PA levels are associated with increased prevalence of steatosis [65, 66]. In contrast, observational and interventional studies demonstrate that higher levels of PA are associated with lower intrahepatic fat content and reduced risk of steatosis, with these effects being independent of changes in body weight. Mechanistically, exercise activates AMP-activated protein kinase and related signalling pathways in hepatocytes, leading to enhanced fatty acid oxidation, increased autophagy and lipophagy and improved mitochondrial function [67]. In addition, the benefits of PA and exercise include increased cardiovascular fitness, and improved systemic endothelial function and muscle health. Exploratory findings suggest that, in the skeletal muscle, exercise promotes secretion of myokines and other exerkines (e.g. irisin and FGF21) that modulate systemic inflammation and improve insulin sensitivity [68]; however, this remains to be confirmed in large and appropriately sized human studies. Moreover, animal models and human translational studies indicate that exercise can remodel hepatic epigenetic marks and gene expression profiles associated with lipid metabolism and inflammatory responses [69, 70]. In addition, exercise was shown to decrease the risk of hepatocellular carcinoma both in animal models [69] and in humans [71] in a dose-dependent manner. Furthermore, PA restores gut microbiota diversity [72], which may contribute to improvements in liver and metabolic health.

In individuals with MASLD, a pilot study suggested an additional benefit of exercise to that obtained by diet and weight loss alone [73]. In this study, regular, unstructured exercise was added to energy restriction, which led to normalisation of ALT levels, while energy restriction alone was not sufficient to achieve this effect [73]. Since then, several studies have been published that confirm this original observation (for details, see electronic supplementary material [ESM] Table 1). Findings of systematic reviews and meta-analyses of studies comparing exercise vs standard of care, or exercise plus diet vs diet only or vs standard of care are summarised in ESM Table 1. As shown, the combination of diet and exercise is superior to diet or exercise alone, but exercise has clear independent benefits vs diet. Importantly, there is no evidence that a single exercise modality is clearly superior to others for reducing liver fat and, provided that sufficient intensity and adherence are met, all modalities (aerobic, resistance, aerobic plus resistance and high-intensity-interval training) are effective and exercise prescription should be individualised according to patient preferences. Combined aerobic and resistance training offers the most comprehensive metabolic benefits [74]. Currently, >150 min/week of moderate-intensity or 75 min/week of vigorous-intensity PA is strongly recommended for both the general population and individuals with MASLD, since it improves liver fat content, liver enzymes, and cardiometabolic risk in MASLD, with benefits being found even without major weight loss [3].

Importantly, in individuals with sarcopenic obesity (particularly in those with cirrhosis) [75], loss of muscle mass with energy-restricted diet is a common clinical problem and this can lead to clinical decompensation. In these individuals, increased protein intake alongside resistance training is required to avoid muscle mass loss [76].

It should be highlighted that bioimpedance devices label ‘non-fat weight loss’ as muscle loss, which is an incorrect assumption since non-fat weight includes water loss (decreased circulating volume and decreased extracellular fluid) [77]. In individuals with type 2 diabetes, non-fat weight loss was not associated with adverse events or a decrease in wellbeing in a 5 year follow-up study on continued support for weight-loss maintenance [78].

## Sleep and circadian rhythm disruption

There is increasing evidence linking poor sleep health with risk of MASLD [79–81]. In a recent dose–response meta-analysis including 31 studies and over 800,000 individuals, the highest sleep-duration categories were associated with lower MASLD risk vs the shortest (OR 0.80 [95% CI 0.74, 0.91]), without a clear dose–response [82]. In a study performed in the NHANES population [83], a U-shaped curve was observed, with the lowest MASLD risk being found with a sleep duration of 7–7.5 h/night. In older adults, <6 h and >8 h sleep were each linked to higher MASLD prevalence (21% and 38% increases, respectively, vs 6–8 h sleep) [84].

Irrespective of sleep duration, a poor sleep pattern (including trouble sleeping, snoring, daytime sleepiness and sleep apnoea) was linked to 3.7-fold higher MASLD risk and higher fibrosis risk [85]. This poor sleep pattern has a synergistic effect with diabetes on liver fibrosis risk in MASLD [86]. Other exploratory findings from studies of circadian rhythm-associated sleep disruption (late bedtimes, irregular schedules, shift work, social jetlag) have been linked to increased incidence of MASLD [87].

Alongside sleep duration, quality and disruption, and regularity of meals (as discussed above), other circadian rhythm-associated lifestyle disruptions might modulate the risk of MASLD [88]. For instance, in a study performed in the UK Biobank prospective population, greater daytime light exposure, reduced nocturnal light exposure and regulated rest–activity rhythms were found as independent protective factors against MASLD and its progression to liver fibrosis and advanced liver disease [89].

## Implementing lifestyle changes to achieve weight loss and MASLD improvement, and sustaining them in the long term

Implementation of lifestyle changes faces major challenges. The well-known limited patient adherence to changes and lack of sustainability of weight loss in the long term exemplify the gap between planning and implementing a lifestyle change with success. For example, in a large cohort of over 2000 overweight or obese individuals with MASLD in standard clinical care who achieved a weight reduction of ≥5%, over 75% regained weight over a median follow-up of 39 months [90]. A large variety of factors may represent barriers to a successful change in dietary and PA habits, including patient awareness, socioeconomic status, lack of social support, healthcare provider communication skills, accessibility and individual variability in response to interventions [91]. In addition, following weight loss, physiological processes oppose further weight loss and make it difficult to maintain the newly achieved body mass; this is likely regulated by genotypic and phenotypic drivers that differ from those acting in the initial phase of weight loss [92].

Some of the best known barriers to sustained positive lifestyle changes can be mitigated by setting quantitative and achievable weight-loss and PA targets, including the reduction of sedentary time, and unstructured and structured exercise (with a real and individualised ‘prescription’ instead of vague suggestion) [93]. In addition, use of techniques such as motivational interview may also be helpful. Tackling this complex issue requires multidisciplinary teams composed of physicians, dietitians, physiotherapists and psychologists that can support a patient’s journey in the long term. Digital tools (e.g. wearables, digital coach) could be implemented to improve patients’ experience and increase adherence to the recommended lifestyle changes [94]. Finally, it is crucial to consider the stage of liver disease in this global assessment, since individuals with cirrhosis, particularly in the decompensated stage, almost universally show sarcopenia and have different needs in terms of macronutrients and safety [95].

## Conclusions and future research

**Fig. 2:**
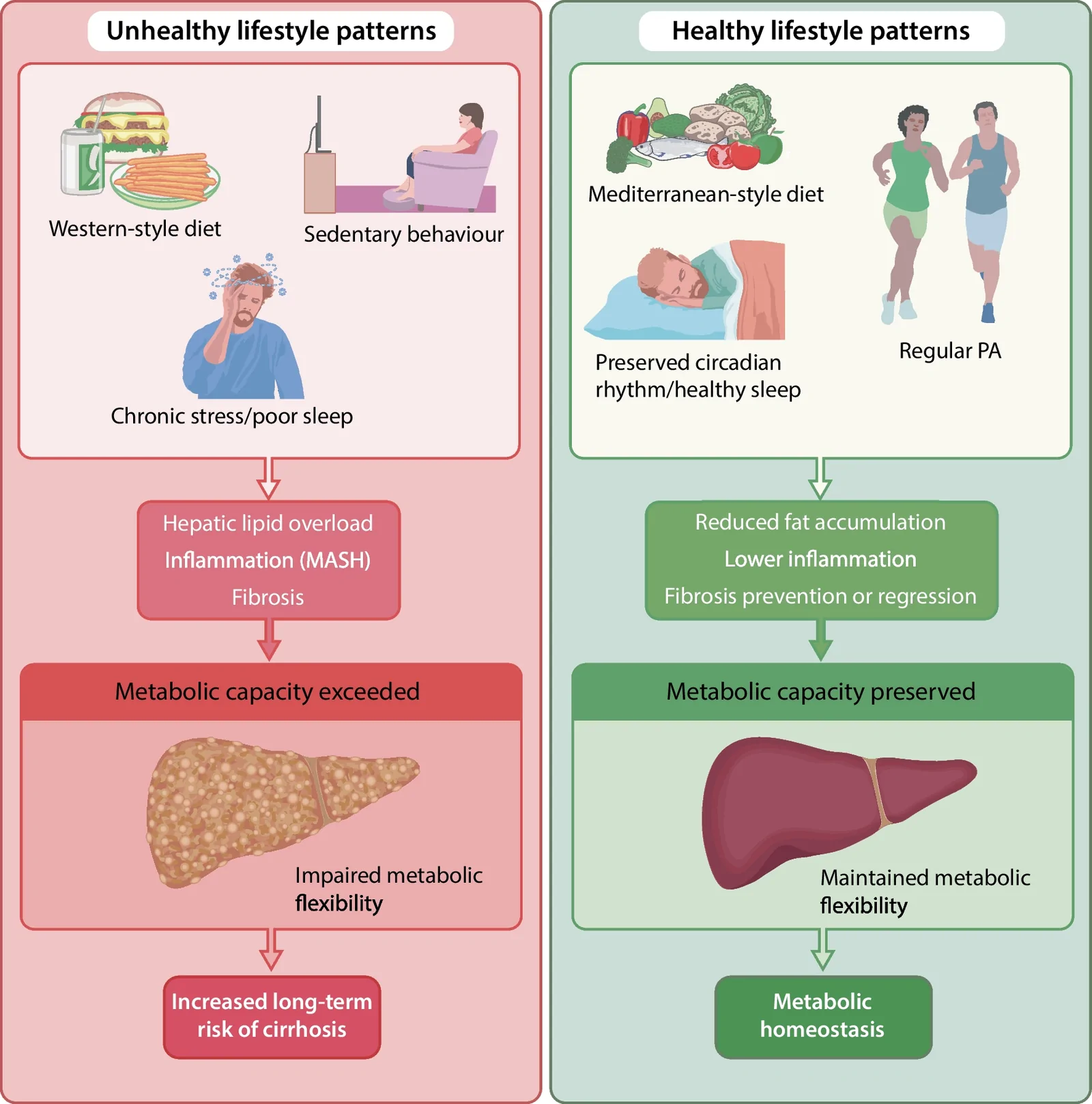
Impact of lifestyle patterns on metabolic capacity and MASLD. Unhealthy lifestyle patterns cause hepatic metabolic capacity to be exceeded and impair metabolic flexibility, promoting hepatic lipid overload, inflammation (MASH), fibrosis and increased long-term risk of cirrhosis. In contrast, healthy lifestyle patterns preserve metabolic capacity and flexibility, reduce hepatic fat accumulation and inflammation, and prevent or reverse fibrosis. This figure is available as part of a downloadable slideset

The effect of lifestyle patterns on weight loss, metabolic reserve and MASLD is summarised in Fig. 2. Weight loss through energy restriction and macronutrient-optimised diet combined with PA remains central in the non-pharmacological management of MASLD [96]. Behavioural interventions (dietary modification, exercising, and optimisation of sleep and circadian habits) exert both weight-loss-dependent and -independent effects [97]. The latter are generally smaller than those achieved with substantial weight reduction but, yet, they are clinically relevant, particularly in non-obese (lean) individuals with MASLD and in individuals who are unable to lose weight or maintain weight loss. Furthermore, phenotypes in MASLD vary considerably (e.g. the presence or absence of type 2 diabetes, severity of obesity and visceral obesity, presence and severity of sarcopenia, sex, age, genetic variants, etc.) [96]. Given this heterogeneity, there may be marked variability in response to behavioural/lifestyle interventions, with some individuals showing clear improvement and others showing negligible effects after the same intervention. Due to this, and since weight loss does not always parallel changes in intrahepatic lipids and inflammation in MASLD, a personalised, multi-modal approach combining optimised lifestyle interventions with targeted pharmacological therapies, such as GLP-1 receptor agonists [98], may be necessary to maximise both weight reduction and direct histological improvement in the liver in non-responders and high-risk phenotypes. It is essential to monitor liver enzymes, steatosis and fibrosis by non-invasive tools (e.g. magnetic resonance imaging-proton density fat fraction [MRI-PDFF] and liver stiffness), and cardiometabolic biomarkers of risk beyond body weight [96]. Future research should aim to provide better knowledge of the genotype–phenotype characterisation of response to behavioural interventions and at identifying biomarkers to individualise the prescription of lifestyle interventions in MASLD. Deep phenotyping using -omics technologies is moving the field closer to this ideal setting [99–103].

## Supplementary Information

Below is the link to the electronic supplementary material.

ESM Table 1 (PDF 253 KB)

Slideset of figures (PPTX 331 KB)

## Abbreviations

ALT: Alanine aminotransferase
FXR: Farnesoid X receptor
LCD: Low-carbohydrate diet
MASLD: Metabolic dysfunction-associated steatotic liver disease
NAFLD: Non-alcoholic fatty liver disease
PA: Physical activity
PUFA: Polyunsaturated fatty acid
SFA: Saturated fatty acid
TGR5: Takeda G-protein-coupled receptor 5

## References

[1] Younossi ZM, Golabi P, Paik JM, Henry A, Van Dongen C, Henry L (2023) The global epidemiology of nonalcoholic fatty liver disease (NAFLD) and nonalcoholic steatohepatitis (NASH): a systematic review. Hepatology 77(4):1335–1347. 10.1097/HEP.0000000000000004 PMC10026948 36626630

[2] Paik JM, Kabbara K, Eberly KE, Younossi Y, Henry L, Younossi ZM (2022) Global burden of NAFLD and chronic liver disease among adolescents and young adults. Hepatology 75(5):1204–1217. 10.1002/hep.32228 34741554

[3] European Association for the Study of the Liver, European Association for the Study of D, European Association for the Study of Obesity (2024) EASL-EASD-EASO Clinical Practice Guidelines on the management of metabolic dysfunction-associated steatotic liver disease (MASLD): executive summary. Diabetologia 67(11):2375–2392. 10.1007/s00125-024-06196-3 PMC11519095 38869512

[4] NCD Collaboration Risk Factor Collaboration (NCD-RisC) (2024) Worldwide trends in underweight and obesity from 1990 to 2022: a pooled analysis of 3663 population-representative studies with 222 million children, adolescents, and adults. Lancet 403(10431):1027–1050. 10.1016/S0140-6736(23)02750-2 PMC7615769 38432237

[5] Bluher M (2025) Understanding the pathophysiology of obesity-the relevance of weight loss strategies through behavior modification. Herz 50(4):233–239. 10.1007/s00059-025-05321-1 40522355

[6] Nauck MA, Tuttle KR, Tschop MH, Bluher M (2026) Glucagon-like receptor agonists and next-generation incretin-based medications: metabolic, cardiovascular, and renal benefits. Lancet 407(10531):892–908. 10.1016/S0140-6736(25)02105-1 41547366

[7] Shea S, Lionis C, Kite C et al (2024) Non-alcoholic fatty liver disease and coexisting depression, anxiety and/or stress in adults: a systematic review and meta-analysis. Front Endocrinol 15:1357664. 10.3389/fendo.2024.1357664 PMC11058984 38689730

[8] Ran S, Zhang J, Tian F et al (2025) Association of metabolic signatures of air pollution with MASLD: observational and Mendelian randomization study. J Hepatol 82(4):560–570. 10.1016/j.jhep.2024.09.033 39349253

[9] Krag A, Tacke F, Israelsen M, Younossi ZM (2026) Is liver steatosis a disease or the emperor’s new clothes? J Hepatol 84(5):993–1001. 10.1016/j.jhep.2025.12.026 41554319

[10] Donnelly KL, Smith CI, Schwarzenberg SJ, Jessurun J, Boldt MD, Parks EJ (2005) Sources of fatty acids stored in liver and secreted via lipoproteins in patients with nonalcoholic fatty liver disease. J Clin Invest 115(5):1343–1351. 10.1172/JCI23621 PMC1087172 15864352

[11] Diraison F, Moulin P, Beylot M (2003) Contribution of hepatic de novo lipogenesis and reesterification of plasma non esterified fatty acids to plasma triglyceride synthesis during non-alcoholic fatty liver disease. Diabetes Metab 29(5):478–485. 10.1016/s1262-3636(07)70061-7 14631324

[12] Petersen KF, Dufour S, Savage DB et al (2007) The role of skeletal muscle insulin resistance in the pathogenesis of the metabolic syndrome. Proc Natl Acad Sci U S A 104(31):12587–12594. 10.1073/pnas.0705408104 PMC1924794 17640906

[13] Fabbrini E, Mohammed BS, Magkos F, Korenblat KM, Patterson BW, Klein S (2008) Alterations in adipose tissue and hepatic lipid kinetics in obese men and women with nonalcoholic fatty liver disease. Gastroenterology 134(2):424–431. 10.1053/j.gastro.2007.11.038 PMC2705923 18242210

[14] Benhamed F, Denechaud PD, Lemoine M et al (2012) The lipogenic transcription factor ChREBP dissociates hepatic steatosis from insulin resistance in mice and humans. J Clin Invest 122(6):2176–2194. 10.1172/JCI41636 PMC3366390 22546860

[15] Shi Y, Cheng KY, Thi TT et al (2026) GPR146 in adipose tissue drives adipose-liver crosstalk and promotes hepatic steatosis in mice. Nat Commun 17(1):3389. 10.1038/s41467-026-70136-5 PMC13065827 41775759

[16] du Plessis J, van Pelt J, Korf H et al (2015) Association of adipose tissue inflammation with histologic severity of nonalcoholic fatty liver disease. Gastroenterology 149(3):635-648 e614. 10.1053/j.gastro.2015.05.044 26028579

[17] Bansal SK, Bansal MB (2024) Pathogenesis of MASLD and MASH - role of insulin resistance and lipotoxicity. Aliment Pharmacol Ther 59(Suppl 1):S10–S22. 10.1111/apt.17930 38451123

[18] Kim H, Nelson P, Nzabarushimana E et al (2025) Multi-omic analysis reveals transkingdom gut dysbiosis in metabolic dysfunction-associated steatotic liver disease. Nat Metab 7(7):1476–1492. 10.1038/s42255-025-01318-6 PMC12320953 40604156

[19] David LA, Maurice CF, Carmody RN et al (2014) Diet rapidly and reproducibly alters the human gut microbiome. Nature 505(7484):559–563. 10.1038/nature12820 PMC3957428 24336217

[20] Camilleri M, Lyle BJ, Madsen KL, Sonnenburg J, Verbeke K, Wu GD (2019) Role for diet in normal gut barrier function: developing guidance within the framework of food-labeling regulations. Am J Physiol Gastrointest Liver Physiol 317(1):G17–G39. 10.1152/ajpgi.00063.2019 PMC6689735 31125257

[21] Zhu S, Zou M, Wu Q et al (2026) The gut-liver axis in metabolic dysfunction-associated steatotic liver disease: from mechanistic insights to precision therapeutics. FASEB J 40(6):e71687. 10.1096/fj.202503607RR PMC12986715 41824007

[22] Osswald A, Wortmann E, Wylensek D et al (2025) Secondary bile acid production by gut bacteria promotes Western diet-associated colorectal cancer. Gut 75(8):1505–1519. 10.1136/gutjnl-2024-332243 PMC13479223 41412727

[23] Pathak P, Liu H, Boehme S et al (2017) Farnesoid X receptor induces Takeda G-protein receptor 5 cross-talk to regulate bile acid synthesis and hepatic metabolism. J Biol Chem 292(26):11055–11069. 10.1074/jbc.M117.784322 PMC5491788 28478385

[24] Shi JS, Wen Y, Wang L et al (2026) An update on bile acid-regulated signalling in the pathogenesis of metabolic dysfunction-associated steatotic liver disease. Liver Int 46(4):e70586. 10.1111/liv.70586 41811003

[25] Thoma C, Day CP, Trenell MI (2012) Lifestyle interventions for the treatment of non-alcoholic fatty liver disease in adults: a systematic review. J Hepatol 56(1):255–266. 10.1016/j.jhep.2011.06.010 21723839

[26] Koutoukidis DA, Koshiaris C, Henry JA et al (2021) The effect of the magnitude of weight loss on non-alcoholic fatty liver disease: a systematic review and meta-analysis. Metabolism 115:154455. 10.1016/j.metabol.2020.154455 33259835

[27] Petersen KF, Dufour S, Befroy D, Lehrke M, Hendler RE, Shulman GI (2005) Reversal of nonalcoholic hepatic steatosis, hepatic insulin resistance, and hyperglycemia by moderate weight reduction in patients with type 2 diabetes. Diabetes 54(3):603–608. 10.2337/diabetes.54.3.603 PMC2995496 15734833

[28] Al-Mrabeh A, Zhyzhneuskaya SV, Peters C et al (2020) Hepatic lipoprotein export and remission of human type 2 diabetes after weight loss. Cell Metab 31(2):233–249. 10.1016/j.cmet.2019.11.018 31866441

[29] Taylor R (2025) Aetiology of type 2 diabetes: an experimental medicine odyssey. Diabetologia 68(7):1375–1389. 10.1007/s00125-025-06428-0 PMC12176950 40316731

[30] Tsompanaki E, Thanapirom K, Papatheodoridi M, Parikh P, Chotai de Lima Y, Tsochatzis EA (2023) Systematic review and meta-analysis: the role of diet in the development of nonalcoholic fatty liver disease. Clin Gastroenterol Hepatol 21(6):1462-1474 e1424. 10.1016/j.cgh.2021.11.026 34838723

[31] Smith RL, Soeters MR, Wust RCI, Houtkooper RH (2018) Metabolic flexibility as an adaptation to energy resources and requirements in health and disease. Endocr Rev 39(4):489–517. 10.1210/er.2017-00211 PMC6093334 29697773

[32] Asghari S, Rezaei M, Rafraf M, Taghizadeh M, Asghari-Jafarabadi M, Ebadi M (2022) Effects of calorie restricted diet on oxidative/antioxidative status biomarkers and serum fibroblast growth factor 21 levels in nonalcoholic fatty liver disease patients: a randomized, controlled clinical trial. Nutrients 14(12):2509. 10.3390/nu14122509 PMC9231395 35745238

[33] Raza S, Rajak S, Yen PM, Sinha RA (2024) Autophagy and hepatic lipid metabolism: mechanistic insight and therapeutic potential for MASLD. NPJ Metab Health Dis 2(1):19. 10.1038/s44324-024-00022-5 PMC11296953 39100919

[34] Hydes TJ, Ravi S, Loomba R, Gray ME (2020) Evidence-based clinical advice for nutrition and dietary weight loss strategies for the management of NAFLD and NASH. Clin Mol Hepatol 26(4):383–400. 10.3350/cmh.2020.0067 PMC7641567 32674529

[35] Steven S, Hollingsworth KG, Al-Mrabeh A et al (2016) Very low-calorie diet and 6 months of weight stability in type 2 diabetes: pathophysiological changes in responders and nonresponders. Diabetes Care 39(5):808–815. 10.2337/dc15-1942 27002059

[36] Shi W, Suo X, Wang Y et al (2025) The associations between irregular breakfast and late-night snacking with metabolic dysfunction-associated steatotic liver disease. Clin Nutr 46:52–59. 10.1016/j.clnu.2025.01.019 39864380

[37] St-Onge MP, Ard J, Baskin ML et al (2017) Meal timing and frequency: implications for cardiovascular disease prevention: a scientific statement from the American heart association. Circulation 135(9):e96–e121. 10.1161/CIR.0000000000000476 PMC8532518 28137935

[38] Ryan MC, Itsiopoulos C, Thodis T et al (2013) The Mediterranean diet improves hepatic steatosis and insulin sensitivity in individuals with non-alcoholic fatty liver disease. J Hepatol 59(1):138–143. 10.1016/j.jhep.2013.02.012 23485520

[39] Westerbacka J, Lammi K, Hakkinen AM et al (2005) Dietary fat content modifies liver fat in overweight nondiabetic subjects. J Clin Endocrinol Metab 90(5):2804–2809. 10.1210/jc.2004-1983 15741262

[40] Luukkonen PK, Sadevirta S, Zhou Y et al (2018) Saturated fat is more metabolically harmful for the human liver than unsaturated fat or simple sugars. Diabetes Care 41(8):1732–1739. 10.2337/dc18-0071 PMC7082640 29844096

[41] Bjermo H, Iggman D, Kullberg J et al (2012) Effects of n-6 PUFAs compared with SFAs on liver fat, lipoproteins, and inflammation in abdominal obesity: a randomized controlled trial. Am J Clin Nutr 95(5):1003–1012. 10.3945/ajcn.111.030114 22492369

[42] George ES, Forsyth A, Itsiopoulos C et al (2018) Practical dietary recommendations for the prevention and management of nonalcoholic fatty liver disease in adults. Adv Nutr 9(1):30–40. 10.1093/advances/nmx007 PMC6333937 29438460

[43] Luukkonen PK, Porthan K, Ahlholm N et al (2023) The PNPLA3 I148M variant increases ketogenesis and decreases hepatic de novo lipogenesis and mitochondrial function in humans. Cell Metab 35(11):1887–1896.e5. 10.1016/j.cmet.2023.10.008 37909034

[44] Fang HL, Lai JT, Lin WC (2008) Inhibitory effect of olive oil on fibrosis induced by carbon tetrachloride in rat liver. Clin Nutr 27(6):900–907. 10.1016/j.clnu.2008.08.004 18824281

[45] Kim SJ, Cho SH, Yun JM (2025) Omega-3 polyunsaturated fatty acids and nonalcoholic fatty liver disease in adults: a meta-analysis of randomized controlled trials. Clin Nutr 50:164–174. 10.1016/j.clnu.2025.05.013 40441053

[46] Ahn J, Jun DW, Lee HY, Moon JH (2019) Critical appraisal for low-carbohydrate diet in nonalcoholic fatty liver disease: review and meta-analyses. Clin Nutr 38(5):2023–2030. 10.1016/j.clnu.2018.09.022 30314924

[47] Thomsen MN, Skytte MJ, Samkani A et al (2025) Replacing dietary carbohydrate with protein and fat improves lipoprotein subclass profile and liver fat in type 2 diabetes independent of body weight: evidence from 2 randomized controlled trials. Am J Clin Nutr 121(2):224–231. 10.1016/j.ajcnut.2024.11.030 39617302

[48] Luukkonen PK, Dufour S, Lyu K et al (2020) Effect of a ketogenic diet on hepatic steatosis and hepatic mitochondrial metabolism in nonalcoholic fatty liver disease. Proc Natl Acad Sci U S A 117(13):7347–7354. 10.1073/pnas.1922344117 PMC7132133 32179679

[49] Maersk M, Belza A, Stodkilde-Jorgensen H et al (2012) Sucrose-sweetened beverages increase fat storage in the liver, muscle, and visceral fat depot: a 6-mo randomized intervention study. Am J Clin Nutr 95(2):283–289. 10.3945/ajcn.111.022533 22205311

[50] Geidl-Flueck B, Hochuli M, Nemeth A et al (2021) Fructose- and sucrose- but not glucose-sweetened beverages promote hepatic de novo lipogenesis: a randomized controlled trial. J Hepatol 75(1):46–54. 10.1016/j.jhep.2021.02.027 33684506

[51] Vos MB, Lavine JE (2013) Dietary fructose in nonalcoholic fatty liver disease. Hepatology 57(6):2525–2531. 10.1002/hep.26299 23390127

[52] Saarinen RH, Virtanen HE, Hantunen S, Salonen JT, Tuomainen TP, Virtanen JK (2026) Associations of fructose consumption with prevalence and incidence of metabolic dysfunction-associated steatotic liver disease-the kuopio ischaemic heart disease risk factor study. J Nutr 156:101318. 10.1016/j.tjnut.2025.101318 PMC12975370 41506326

[53] Lustig RH (2013) Fructose: it’s “alcohol without the buzz.” Adv Nutr 4(2):226–235. 10.3945/an.112.002998 PMC3649103 23493539

[54] Faienza MF, Baima J, Cecere V et al (2025) Fructose intake and unhealthy eating habits are associated with MASLD in pediatric obesity: a cross-sectional pilot study. Nutrients 17(4):631. 10.3390/nu17040631 PMC11858415 40004960

[55] Schwimmer JB, Ugalde-Nicalo P, Welsh JA et al (2019) Effect of a low free sugar diet vs usual diet on nonalcoholic fatty liver disease in adolescent boys: a randomized clinical trial. JAMA 321(3):256–265. 10.1001/jama.2018.20579 PMC6440226 30667502

[56] Kechagias S, Ernersson A, Dahlqvist O et al (2008) Fast-food-based hyper-alimentation can induce rapid and profound elevation of serum alanine aminotransferase in healthy subjects. Gut 57(5):649–654. 10.1136/gut.2007.131797 PMC2565580 18276725

[57] Yin HY, You QH, Zhang WJ, Ji G, Dang YQ (2026) High-protein diets and metabolic dysfunction-associated steatotic liver disease: a double-edged sword in liver health. World J Gastroenterol 32(6):113804. 10.3748/wjg.v32.i6.113804 PMC12898319 41695277

[58] Markova M, Pivovarova O, Hornemann S et al (2017) Isocaloric diets high in animal or plant protein reduce liver fat and inflammation in individuals with type 2 diabetes. Gastroenterology 152(3):571-585 e578. 10.1053/j.gastro.2016.10.007 27765690

[59] Zelber-Sagi S, Ivancovsky-Wajcman D, FlissIsakov N et al (2018) High red and processed meat consumption is associated with non-alcoholic fatty liver disease and insulin resistance. J Hepatol 68(6):1239–1246. 10.1016/j.jhep.2018.01.015 29571924

[60] Cantero I, Abete I, Monreal JI, Martinez JA, Zulet MA (2017) Fruit fiber consumption specifically improves liver health status in obese subjects under energy restriction. Nutrients 9(7):667. 10.3390/nu9070667 PMC5537782 28657604

[61] Dranoff JA (2023) Coffee as chemoprotectant in fatty liver disease: caffeine-dependent and caffeine-independent effects. Am J Physiol Gastrointest Liver Physiol 324(6):G419–G421. 10.1152/ajpgi.00026.2023 36976807

[62] Chen YP, Lu FB, Hu YB, Xu LM, Zheng MH, Hu ED (2019) A systematic review and a dose-response meta-analysis of coffee dose and nonalcoholic fatty liver disease. Clin Nutr 38(6):2552–2557. 10.1016/j.clnu.2018.11.030 30573353

[63] Ebadi M, Ip S, Bhanji RA, Montano-Loza AJ (2021) Effect of coffee consumption on non-alcoholic fatty liver disease incidence, prevalence and risk of significant liver fibrosis: systematic review with meta-analysis of observational studies. Nutrients 13(9):3042. 10.3390/nu13093042 PMC8471033 34578919

[64] Kakiyama G, Minowa K, Rodriguez-Agudo D et al (2022) Coffee modulates insulin-hepatocyte nuclear factor-4alpha-Cyp7b1 pathway and reduces oxysterol-driven liver toxicity in a nonalcoholic fatty liver disease mouse model. Am J Physiol Gastrointest Liver Physiol 323(5):G488–G500. 10.1152/ajpgi.00179.2022 PMC9639758 36193897

[65] van Kleef LA, Hofman A, Voortman T, de Knegt RJ (2022) Objectively measured physical activity is inversely associated with nonalcoholic fatty liver disease: the Rotterdam Study. Am J Gastroenterol 117(2):311–318. 10.14309/ajg.0000000000001584 PMC8812433 34904966

[66] Kim D, Vazquez-Montesino LM, Li AA, Cholankeril G, Ahmed A (2020) Inadequate physical activity and sedentary behavior are independent predictors of nonalcoholic fatty liver disease. Hepatology 72(5):1556–1568. 10.1002/hep.31158 32012316

[67] Berzigotti A, Saran U, Dufour JF (2016) Physical activity and liver diseases. Hepatology 63(3):1026–1040. 10.1002/hep.28132 26313307

[68] Safdar A, Saleem A, Tarnopolsky MA (2016) The potential of endurance exercise-derived exosomes to treat metabolic diseases. Nat Rev Endocrinol 12(9):504–517. 10.1038/nrendo.2016.76 27230949

[69] Piguet AC, Saran U, Simillion C et al (2015) Regular exercise decreases liver tumors development in hepatocyte-specific PTEN-deficient mice independently of steatosis. J Hepatol 62(6):1296–1303. 10.1016/j.jhep.2015.01.017 25623824

[70] Stevanovic J, Beleza J, Coxito P, Ascensao A, Magalhaes J (2020) Physical exercise and liver “fitness”: role of mitochondrial function and epigenetics-related mechanisms in non-alcoholic fatty liver disease. Mol Metab 32:1–14. 10.1016/j.molmet.2019.11.015 PMC6931125 32029220

[71] DiJoseph K, Thorp A, Harrington A, Schmitz KH, Chinchilli VM, Stine JG (2023) Physical activity and risk of hepatocellular carcinoma: a systematic review and meta-analysis. Dig Dis Sci 68(3):1051–1059. 10.1007/s10620-022-07601-w 35767138

[72] Cheng R, Wang L, Le S et al (2022) A randomized controlled trial for response of microbiome network to exercise and diet intervention in patients with nonalcoholic fatty liver disease. Nat Commun 13(1):2555. 10.1038/s41467-022-29968-0 PMC9091228 35538056

[73] Ueno T, Sugawara H, Sujaku K et al (1997) Therapeutic effects of restricted diet and exercise in obese patients with fatty liver. J Hepatol 27(1):103–107. 10.1016/s0168-8278(97)80287-5 9252081

[74] Lindstad TM, Pourteymour S, Lee-Odegard S, Drevon CA, Norheim FA (2026) Structured exercise interventions and hepatic-metabolic outcomes in adults with MASLD: a narrative review of randomized controlled trials. Int J Mol Sci 27(7):2941. 10.3390/ijms27072941 PMC13074135 41977131

[75] Kim G, Lee SE, Lee YB et al (2018) Relationship between relative skeletal muscle mass and nonalcoholic fatty liver disease: a 7-year longitudinal study. Hepatology 68(5):1755–1768. 10.1002/hep.30049 29679374

[76] European Association for the Study of the Liver (2019) EASL Clinical Practice Guidelines on nutrition in chronic liver disease. J Hepatol 70(1):172–193. 10.1016/j.jhep.2018.06.024 PMC6657019 30144956

[77] Tinsley GM, Heymsfield SB (2024) Fundamental body composition principles provide context for fat-free and skeletal muscle loss with GLP-1 RA treatments. J Endocr Soc 8(11):bvae164. 10.1210/jendso/bvae164 PMC11450469 39372917

[78] Lean ME, Leslie WS, Barnes AC et al (2024) 5-year follow-up of the randomised Diabetes Remission Clinical Trial (DiRECT) of continued support for weight loss maintenance in the UK: an extension study. Lancet Diabetes Endocrinol 12(4):233–246. 10.1016/S2213-8587(23)00385-6 38423026

[79] Perez-Diaz-Del-Campo N, Castelnuovo G, Caviglia GP, Armandi A, Rosso C, Bugianesi E (2022) Role of circadian clock on the pathogenesis and lifestyle management in non-alcoholic fatty liver disease. Nutrients 14(23):5053. 10.3390/nu14235053 PMC9736115 36501083

[80] Marjot T, Smith K, Westcott F et al (2026) Human MASLD is a diurnal disease driven by multisystem insulin resistance and reduced insulin availability at night. Cell Metab 38(3):474-492 e476. 10.1016/j.cmet.2025.12.004 PMC7619395 41529695

[81] Marjot T, Tomlinson JW, Hodson L, Ray DW (2023) Timing of energy intake and the therapeutic potential of intermittent fasting and time-restricted eating in NAFLD. Gut 72(8):1607–1619. 10.1136/gutjnl-2023-329998 PMC10359613 37286229

[82] Pirahesh K, Zarrinnia A, Nikniaz L, Nikniaz Z (2025) Association between sleep duration and risk of nonalcoholic fatty liver disease: a systematic review and meta-analysis. Prev Med Rep 50:102968. 10.1016/j.pmedr.2025.102968 PMC11783128 39897736

[83] Wang D, Zhang X, Cai Y, Dong H, Zhang Y (2025) Multidimensional sleep impairment predicts steatotic liver disease spectrum risk. Sci Rep 15(1):10405. 10.1038/s41598-025-95336-9 PMC11947187 40140484

[84] Zhang F, Xue Y, Li W (2025) Exploring the impact of sleep duration and sleep disorders on metabolic dysfunction-associated steatotic liver disease in older adults. BMC Geriatr 25(1):444. 10.1186/s12877-025-06115-0 PMC12219953 40604444

[85] Li Y, Tan S (2024) Sleep factors were associated with a higher risk of MAFLD and significant fibrosis. Sleep Breath 28(3):1381–1391. 10.1007/s11325-024-03017-0 38514588

[86] Zhang C, Cao L, Xu B, Zhang W (2024) Interaction between trouble sleeping and diabetes on metabolic dysfunction-associated fatty liver disease and liver fibrosis in adults results from the National Health and Nutrition Examination Survey 2017–2018. Eur J Gastroenterol Hepatol 36(12):1437–1446. 10.1097/MEG.0000000000002860 PMC11527372 39373628

[87] Shetty A, Hsu JW, Manka PP, Syn WK (2018) Role of the circadian clock in the metabolic syndrome and nonalcoholic fatty liver disease. Dig Dis Sci 63(12):3187–3206. 10.1007/s10620-018-5242-x 30121811

[88] Manoogian EN, Panda S (2016) Circadian clock, nutrient quality, and eating pattern tune diurnal rhythms in the mitochondrial proteome. Proc Natl Acad Sci U S A 113(12):3127–3129. 10.1073/pnas.1601786113 PMC4812772 26979954

[89] Wu H, Wang W, Weng B et al (2025) Diurnal light exposure and rest-activity rhythms in relation to MASLD: insights from two nationwide cohort studies. J Clin Endocrinol Metab 111(5):1459–1469. 10.1210/clinem/dgaf59 PMC13099217 41160717

[90] Malespin MH, Barritt AST, Watkins SE et al (2022) Weight loss and weight regain in usual clinical practice: results from the TARGET-NASH observational cohort. Clin Gastroenterol Hepatol 20(10):2393-2395 e2394. 10.1016/j.cgh.2021.01.023 33486083

[91] Hernandez Roman J, Patel S (2020) Why do lifestyle recommendations fail in most patients with nonalcoholic fatty liver disease? Gastroenterol Clin North Am 49(1):95–104. 10.1016/j.gtc.2019.10.001 32033766

[92] Flanagan EW, Spann R, Berry SE et al (2023) New insights in the mechanisms of weight-loss maintenance: summary from a Pennington symposium. Obesity 31(12):2895–2908. 10.1002/oby.23905 PMC10915908 37845825

[93] Hallsworth K, Adams LA (2019) Lifestyle modification in NAFLD/NASH: Facts and figures. JHEP Rep 1(6):468–479. 10.1016/j.jhepr.2019.10.008 PMC7005657 32039399

[94] Kravchuk S, Bychkov M, Kozyk M, Strubchevska O, Kozyk A (2025) Managing Metabolic Dysfunction-Associated Steatotic Liver Disease (MASLD) in the digital era: overcoming barriers to lifestyle change. Cureus 17(5):e84803. 10.7759/cureus.84803 PMC12188028 40568294

[95] Tandon P, Ismond KP, Riess K et al (2018) Exercise in cirrhosis: translating evidence and experience to practice. J Hepatol 69(5):1164–1177. 10.1016/j.jhep.2018.06.017 29964066

[96] European Association for the Study of the Liver, European Association for the Study of D, European Association for the Study of O (2024) EASL-EASD-EASO Clinical Practice Guidelines on the management of metabolic dysfunction-associated steatotic liver disease (MASLD). J Hepatol 81(3):492–542. 10.1016/j.jhep.2024.04.031 38851997

[97] Keating SE, Chawla Y, De A, George ES (2024) Lifestyle intervention for metabolic dysfunction-associated fatty liver disease: a 24-h integrated behavior perspective. Hepatol Int 18(Suppl 2):959–976. 10.1007/s12072-024-10663-9 PMC11450077 38717691

[98] Newsome PN, Buchholtz K, Cusi K et al (2021) A placebo-controlled trial of subcutaneous semaglutide in nonalcoholic steatohepatitis. N Engl J Med 384(12):1113–1124. 10.1056/NEJMoa2028395 33185364

[99] Raverdy V, Tavaglione F, Chatelain E et al (2024) Data-driven cluster analysis identifies distinct types of metabolic dysfunction-associated steatotic liver disease. Nat Med 30(12):3624–3633. 10.1038/s41591-024-03283-1 PMC11645276 39653777

[100] Ma X, Ding L, Li S et al (2025) Druggable genome-wide Mendelian randomization identifies therapeutic targets for metabolic dysfunction-associated steatotic liver disease. Lipids Health Dis 24(1):113. 10.1186/s12944-025-02515-8 PMC11938603 40140823

[101] Jamialahmadi O, De Vincentis A, Tavaglione F et al (2024) Partitioned polygenic risk scores identify distinct types of metabolic dysfunction-associated steatotic liver disease. Nat Med 30(12):3614–3623. 10.1038/s41591-024-03284-0 PMC11645285 39653778

[102] MoTr PACSG, Lead A, MoTr PACSG (2024) Temporal dynamics of the multi-omic response to endurance exercise training. Nature 629(8010):174–183. 10.1038/s41586-023-06877-w PMC11062907 38693412

[103] Hu CR, Zeng CF, Li H et al (2026) Multi-omics analysis of the gut-liver axis revealed the effects of different exercise interventions in NAFLD mice. BMC Microbiol 26(1):254. 10.1186/s12866-026-04711-7 PMC12998337 41673803

[104] Johari MI, Yusoff K, Haron J et al (2019) A randomised controlled trial on the effectiveness and adherence of modified alternate-day calorie restriction in improving activity of non-alcoholic fatty liver disease. Sci Rep 9(1):11232. 10.1038/s41598-019-47763-8 PMC6677794 31375753

[105] Wei X, Lin B, Huang Y et al (2023) Effects of time-restricted eating on nonalcoholic fatty liver disease: the TREATY-FLD randomized clinical trial. JAMA Netw Open 6(3):e233513. 10.1001/jamanetworkopen.2023.3513 PMC10024204 36930148

[106] Sun X, Li F, Yan H et al (2025) Intermittent compared with continuous calorie restriction for treatment of metabolic dysfunction-associated steatotic liver disease: a randomized clinical trial. Am J Clin Nutr 121(1):158–166. 10.1016/j.ajcnut.2024.10.012 39447676

[107] Wang YY, Tian F, Qian XL, Ying HM, Zhou ZF (2024) Effect of 5:2 intermittent fasting diet versus daily calorie restriction eating on metabolic-associated fatty liver disease-a randomized controlled trial. Front Nutr 11:1439473. 10.3389/fnut.2024.1439473 PMC11368853 39229586

[108] Lee HA, Moon H, Kim Y, Lee JK, Lee HA, Kim HY (2025) Effects of intermittent calorie restriction in nondiabetic patients with metabolic dysfunction-associated steatotic liver disease. Clin Gastroenterol Hepatol 23(1):114-123 e113. 10.1016/j.cgh.2024.06.051 39181426

[109] Oh JH, Yoon EL, Park H et al (2025) Efficacy and safety of time-restricted eating in metabolic dysfunction-associated steatotic liver disease. J Hepatol 83(6):1256–1265. 10.1016/j.jhep.2025.06.005 40543603

[110] Nilghaz M, Sadeghi A, Koochakpoor G et al (2025) The efficacy of DASH combined with time-restricted feeding (16/8) on metabolic associated fatty liver disease management: a randomized controlled trial. Sci Rep 15(1):7020. 10.1038/s41598-025-88393-7 PMC11868424 40016311

[111] Shafiee M, Sadeghi A, Ghafouri-Taleghani F et al (2025) Effects of time restricted feeding combined with Lacto Ovo vegetarian diet on metabolic associated fatty liver disease management: a randomized clinical trial. Sci Rep 15(1):4463. 10.1038/s41598-025-88773-z PMC11803106 39915600

[112] OzluKarahan T, Yilmaz Akyuz E, Yilmaz Karadag D, Yilmaz Y, Eren F (2025) Effects of intermittent fasting on liver steatosis and fibrosis, serum FGF-21 and autophagy markers in metabolic dysfunction-associated fatty liver disease: a randomized controlled trial. Life 15(5):696. 10.3390/life15050696 PMC12113254 40430125

