# Supplementary material for: Non-pharmacological treatment of MASLD
Source: Diabetologia. 2026 Jul 25;69(11):3162–74. doi: 10.1007/s00125-026-06811-5 (PMC13627214; doi:10.1007/s00125-026-06811-5)
Supplement: Supplementary file 1 [file 125_2026_6811_MOESM1_ESM.pdf]

ESM Table 1. Systematic reviews and meta-analysis analyzing the effect of exercise alone or in combination with diet in MASLD. Abbreviations: standardized mean difference (SMD); intrahepatic lipids (IHL).

**Intervention comparison**	**Main hepatic and metabolic effects**	**Key quantitative findings**	**Major conclusions**
Exercise alone vs control (1-5)	Exercise reduces liver enzymes and markedly reduces IHL	Aerobic exercise reduced IHL (SMD −0.98); ALT, AST, weight and BMI vs control<br>High intensity intermittent training reduces IHL in patients with type 2 diabetes.	Exercise can lower liver fat even without major weight loss
Exercise vs diet vs combined (1, 6-9)	All approaches improve liver parameters; combined intervention consistently best	Diet-only and exercise-only improve ALT/AST and IHL; combined yields larger ALT, AST, HOMA-IR and IHTG changes	Diet and exercise are complementary rather than interchangeable
Exercise + diet vs control<br>(1, 6, 7) (8, 9)	Largest and most consistent improvements in liver enzymes, steatosis and insulin resistance	ALT MD −13.27; HOMA-IR MD −2.07; ~50% IHL reduction vs ~30% with exercise alone	Strong evidence that combining diet and exercise is superior to either alone
Exercise modality comparison<br>(10) (11)	All exercise modes beneficial; modality-specific effects on enzymes and lipids	Network MA: aerobic+resistance best for dyslipidemia; aerobic training best for ALT; resistance training best for AST; high intensity interval training reduces IHL	No single modality clearly superior for IHL; choice can be individualized

References to ESM Table 1

1. Katsagoni CN, Georgoulis M, Papatheodoridis GV, Panagiotakos DB, Kontogianni MD. Effects of lifestyle interventions on clinical characteristics of patients with non-alcoholic fatty liver disease: A meta-analysis. Metabolism. 2017;68:119–32.
2. Wang ST, Zheng J, Peng HW, Cai XL, Pan XT, Li HQ, et al. Physical activity intervention for non-diabetic patients with non-alcoholic fatty liver disease: a meta-analysis of randomized controlled trials. BMC Gastroenterol. 2020;20(1):66.
3. Barron-Cabrera E, Soria-Rodriguez R, Amador-Lara F, Martinez-Lopez E. Physical Activity Protocols in Non-Alcoholic Fatty Liver Disease Management: A Systematic Review of Randomized Clinical Trials and Animal Models. Healthcare (Basel). 2023;11(14).
4. Cassidy S, Thoma C, Hallsworth K, Parikh J, Hollingsworth KG, Taylor R, et al. High intensity intermittent exercise improves cardiac structure and function and reduces liver fat in patients with type 2 diabetes: a randomised controlled trial. Diabetologia. 2016;59(1):56–66.
5. Hallsworth K, Fattakhova G, Hollingsworth KG, Thoma C, Moore S, Taylor R, et al. Resistance exercise reduces liver fat and its mediators in non-alcoholic fatty liver disease independent of weight loss. Gut. 2011;60(9):1278–83.
6. Golabi P, Locklear CT, Austin P, Afdhal S, Byrns M, Gerber L, et al. Effectiveness of exercise in hepatic fat mobilization in non-alcoholic fatty liver disease: Systematic review. World J Gastroenterol. 2016;22(27):6318–27.
7. Kenneally S, Sier JH, Moore JB. Efficacy of dietary and physical activity intervention in non-alcoholic fatty liver disease: a systematic review. BMJ Open Gastroenterol. 2017;4(1):e000139.
8. Fernandez T, Vinuela M, Vidal C, Barrera F. Lifestyle changes in patients with non-alcoholic fatty liver disease: A systematic review and meta-analysis. PLoS One. 2022;17(2):e0263931.
9. Houttu V, Csader S, Nieuwdorp M, Holleboom AG, Schwab U. Dietary Interventions in Patients With Non-alcoholic Fatty Liver Disease: A Systematic Review and Meta-Analysis. Front Nutr. 2021;8:716783.
10. Xue Y, Peng Y, Zhang L, Ba Y, Jin G, Liu G. Effect of different exercise modalities on nonalcoholic fatty liver disease: a systematic review and network meta-analysis. Sci Rep. 2024;14(1):6212.
11. Fu J, Liu C, Yang L, Zhang B, Zhou R, Deng C, et al. Effect of high-intensity interval training on clinical parameters in patients with metabolic dysfunction-associated steatotic liver disease: a systematic review and meta-analysis of randomized controlled trials. Eur J Gastroenterol Hepatol. 2025;37(7):789–98.
